# Supplementary material for: Discrete long-range on-cell motion of bacteriophage T4
Source: Microbiol Spectr. 2025 Dec 17;14(2):e02509-25. doi: 10.1128/spectrum.02509-25 (PMC12889154; doi:10.1128/spectrum.02509-25)
Supplement: Supplemental material — Fig. S1 to S14; Table S1. [file spectrum.02509-25-s0001.pdf]

Supplementary Information

Discrete long-range on-cell motion of bacteriophage T4

Lisa Laura Dreesens[1], Igor Rutka[1], Kyriacos Nicolaou[1], Marie-Eve Aubin-Tam[1#]

[1]Department of Bionanoscience, Kavli Institute of Nanoscience, Delft University of Technology, Netherlands.

[#] correspondence to Marie-Eve Aubin-Tam: m.e.aubin-tam@tudelft.nl

Supplementary 1

The average trajectory duration of *free* phages was 0.88 seconds. Symmetric instantaneous velocity distribution graph showed that *x* and *y* displacements had mean values centered around 0 (Supplementary figure 6A). Further, the normalized velocity autocorrelation showed, with the exception of $\Delta t = 0$, a mean value centered around 0 during the course of time (Supplementary figure 6B). This confirms the absence of apparent flow during imaging of phages within the flow cell. The diffusion coefficient calculated for fluorescently labeled T4 phage particles (Supplementary figure 6C-D) approximates a spherical particle with a radius of 85 nm, which is in close agreement with the size that we measured by dynamic light scattering (DLS), i.e. R_h of 78.7 ± 0.8 nm (Mean ± SD) for labeled phage T4 and 77.83 ± 0.35 nm (Mean ± SD) for the control group (i.e., unlabeled phage T4). The DLS measurements to estimate hydrodynamic radius were obtained using DynaPro NanoStar, with each measurement representing the average of 20 acquisitions performed at RT. Even though T4 phage particles have a more ellipsoid shape, the indicative size estimated from free diffusion was also relatively close to that for T4 phage particles observed by EM with LTF in the retracted position (dimensions of head, sheath and baseplate are 115 nm x 85 nm, 93 nm x 24 nm, and 27 nm x 52 nm respectively [1, 2]), confirming that each particle indeed presented intact individual phages. The obtained empirical cumulative distribution function (CDF) was tested, using a Kolmogorov-Smirnov test, against the null-hypothesis of a standard normal CDF. These results showed that the obtained α values of *free* T4 phage trajectories in this set-up did not deviate significantly from a standard Gaussian distribution (Kolmogorov-Smirnov test, $p = 0.14$; Supplementary figure 6E). This demonstrates that labeled T4 phages within this set-up exhibited a normal (Fickian) diffusion consistent with free diffusion, pure Brownian motion, in bulk.

Supplementary 2

We inquired whether the observed motion of the *interacting* phage trajectories could originate from movements of the flow cell with respect to the camera (microscope stage drift), movement of the cell as result from sub-optimal cell adhesion to the flow cell surface, or diffusion of host receptors at the cell surface. To investigate this, we quantified the rates of phage motion in 10 representative focal trajectories of the class of *interacting* phage trajectories (Supplementary figure 10). These focal trajectories met the following criteria: i) exceeded the theoretical area accessible by a phage tethered to a single point on the cell surface (showed a displacement of more than 540 nm), ii) phage position during cell-interaction could be identified and tracked for each consecutive frame, iii) the trajectory was obtained at host cells for which the cell outline could reliably be detected by the cell-motion algorithm, iv) and contained *bound* phages (immobilized points) within the same field of view to

ensure exclusion of motion that has occurred through drift of image plane during imaging of the *interacting* phage trajectory.

We quantified the microscope stage drift and motion of the cells with which the focal phages interacted in the imaging plane. We tracked the mid-point position of four cross-sections (three on the short and one on the long axis) over time for each of these cells, using another cell movement analysis with a higher resolution. This allowed us to quantitatively analyze the amount of i) translational motion (i.e., drift), ii) subtle rotational motion (i.e., cell movement), and iii) noise (i.e., phase shifts of areas outside the focal plane giving rise to out-of-focus blur and optical phase contrast artifacts e.g., halos and shade-off contrast patterns, causing variations in the intensity gradient impairing uncertainty in the quantification of intensity and therefore position of a particle) with subpixel level accuracy.

Here, the phase contrast image of the cell was subjected to a Gaussian filter with sigma 1 and SD of 1 pixel, followed by a cubic interpolation and a threshold based on the Otsu method [3]. Using the open-source Python package skimage [4], the cell was identified by defining the image objects properties using the function regionprops, followed by determining the long axis of the cell (L_x). The cross-sections along the short axis of the cell (S_x) were defined by lines perpendicular to the L_x. The first cross section was set at the midpoint of the cell, with the two remaining cross-sections halfway between the poles and mid-cell. The mid-point of the cell within each of the cross-sections was determined by fitting Equation 1 to the intensity profile of the cross-section, with *f(x)* the intensity function, *x* the coordinate along the slice, *σ* the width of the cell, and μ the mid-point of the cell:

$$f(x) = e^{\left(\frac{x-\mu}{2\sigma}\right)^n} \qquad \text{Equation 1}$$

The *n* was set to either 4 or 6, based on the highest obtained quality of the fit (R^2). To define the region of the cell to which this function was fitted, the intensity profile along the cross-section was divided by two. The maximal intensity point within each of these regions was determined and set as the outer boundary of the intensity profile. A transformation on the intensity values was applied by subtracting the highest measured intensity value (*I*) from each of the remaining pixels within the cross-section, followed by inversion, as seen in Equation 2:

$$I' = max(I) - I \qquad \text{Equation 2}$$

The intensity profile was normalized to 1, based on the maximal detected intensity value as stated in Equation 3:

$$I'' = \frac{I'}{max\,(I')} \qquad \text{Equation 3}$$

Using this procedure, we tracked the coordinates for the mid-point position along the four cross-sections of each cell over time to assess subtle rotational and translational movements at subpixel level accuracy.

Results of the cell-motion analysis showed that translational motion (microscope stage drift) occurred at rates of 2.97 ± 2.21 and 3.54 ± 2.85 nm s^{-1} (mean ± SD, n = 10) in x and y, respectively. While the phage's average speed of the apparently unconstrained on-cell motion was 30 ± 18 nm s^{-1} (mean ± SD, n = 10). This value was obtained by calculating a conservative rate, the maximal distance between two points within the convex hull of the tethered segment divided by the corresponding duration. The obtained rate for the phage's on-cell motion was significantly larger than the measured rate of stage drift (n = 10, p < 0.0001). A lack of translational motion due to microscope stage drift was also corroborated by tightly bound phages present in the same field of view.

Rotational motion was not detectable for phage trajectories imaged at 50 Hz, but only detected for phage trajectories obtained at 200 Hz (4 cells), revealing a maximal amplitude of 12 nm and average period of 7 seconds. These values are, respectively, extremely slow and small compared to our conservative estimate of the speed of the unconstrained long-range on-cell component of the phage trajectories and therefore deemed negligible to our observations.

In addition, we assessed the noise level of the microscope set-up and therefore the lower detection limit. We found that the midpoint position of these focal cells (n = 10) fluctuated on average less than 13 nm between frames. We detected that FTF values remain within 25 nm of the average mid-point position. This indicates that the noise level causing fluctuation in detection of the cell's position remained within a maximum range of 50 nm. While these fast FTF fluctuations could have contributed to the observed motion of tightly bound phages around a single mean point of tethering location, it does not explain the less constrained and longer-distance motion observed for some *interacting* phage trajectories.

Lastly, we considered receptor movement as an explanatory factor. Based on the low rate of receptor drift measured by Ghosh and Young [5] on the timescale of our obtained trajectories (i.e., no visual receptor movement on the timescale of minutes) receptor motion as an explanatory factor is deemed unlikely.

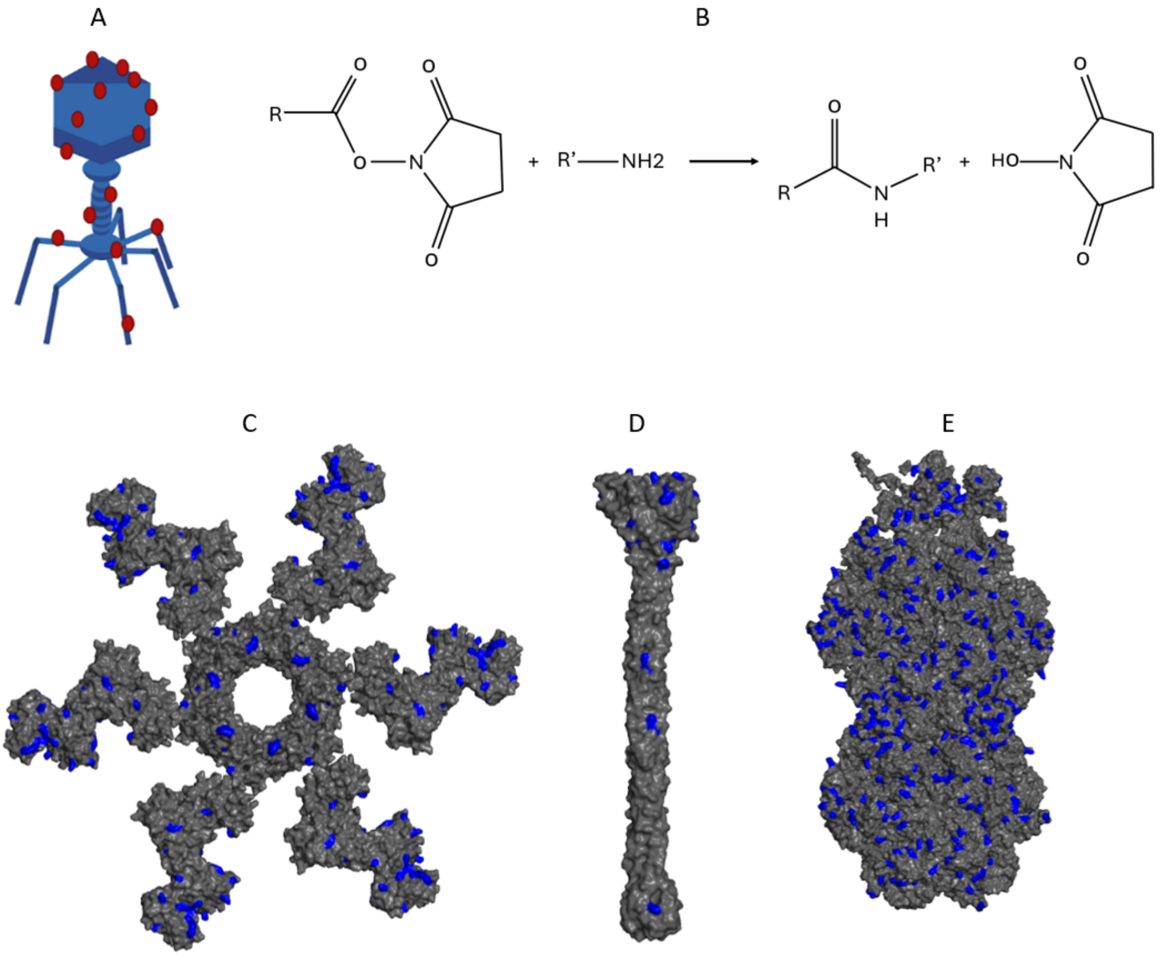

Supplementary figure 1 Fluorescent labeling strategy of bacteriophage T4. A, Phage T4 can be fluorescently labeled with chemical conjugation of surface exposed amino acids with corresponding reactive groups bound to a dye molecule. **B**, NHS-ester reaction scheme for chemical conjugation of dye molecules to phage T4. Here the dye molecule (R) containing an NHS ester reacts with a primary amine group or N-terminus that is exposed on the outside of the protein coat of the phage (R'). A stable amine bond is formed between the dye and the phage, leaving an NHS group as a byproduct. Phage T4 has many exposed lysine residues that provide potential labeling sites for chemical conjugation with dyes containing an NHS-ester group. Lysines (blue) marked on **C**, the sheath (top view), **D**, the distal end of the LTF (side view), and **E**, a single capsomer consisting of gp24 pentamer and gp23 hexamer (side view).

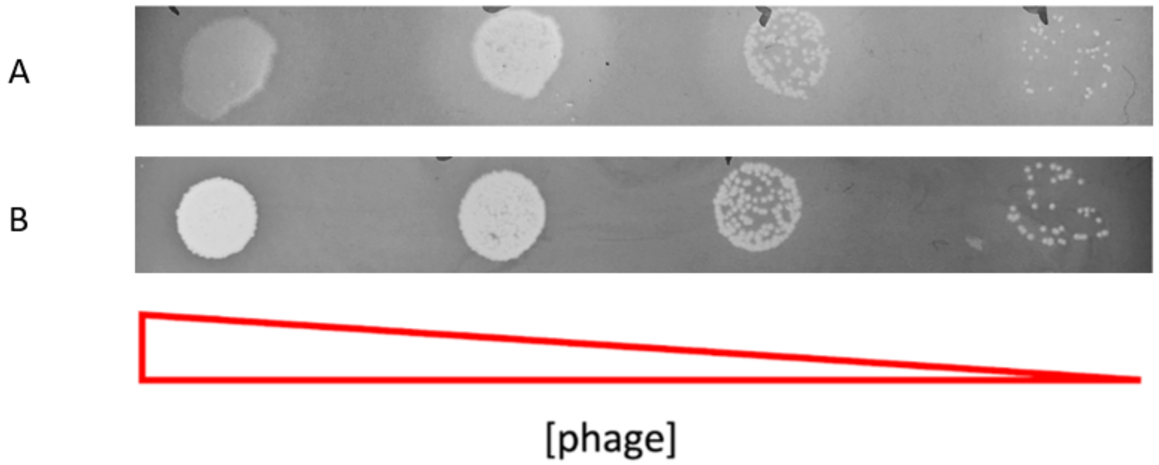

Supplementary figure 2 Effect of labeling on the active titer of T4 phages. Spot assays of increasingly diluted unlabeled (**A**) and Alexa647 labeled (**B**) T4 phage on *E. coli* B. Small clearance zones in bacterial lawn represent plaques formed by individual particles.

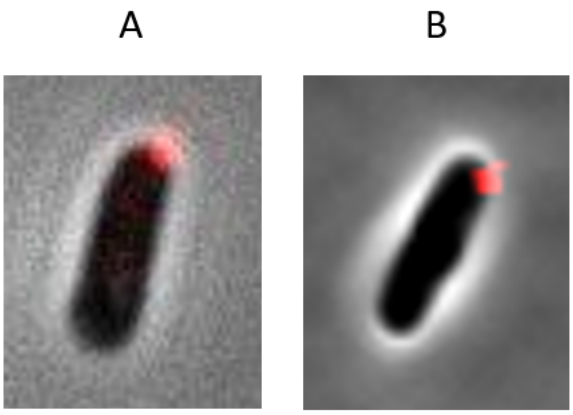

Supplementary figure 3 Irreversible binding of fluorescently labeled T4 phage to **A**, *E. coli* B and **B**, K12.

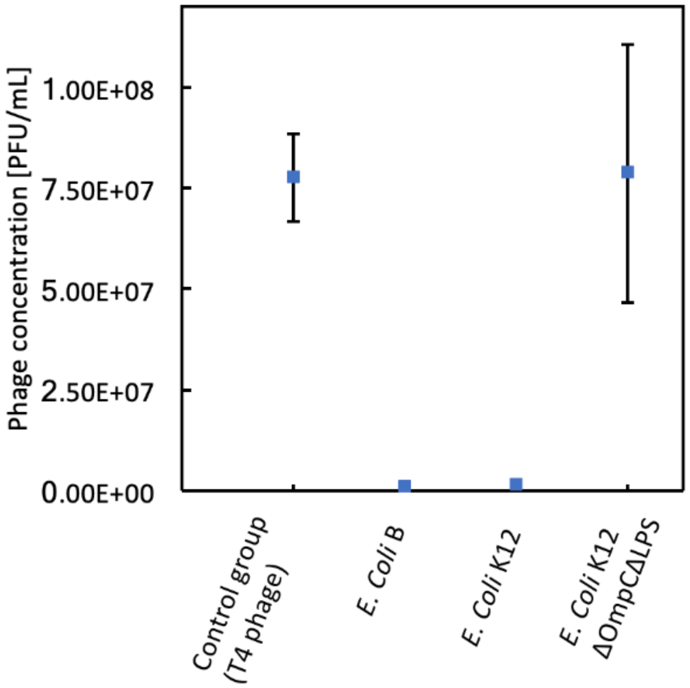

Supplementary figure 4 Effect of presence of host receptor binding proteins on irreversible binding efficiency of phage T4. Irreversible bulk binding experiment of phage T4 with *E. coli* B, *E. coli* K12 and *E. coli* K12 ΔOmpCΔLPS. *Y*-axis represents the concentration of phages that did not bind the host. Data of the control group (T4 phage) represents 2 replicas and those incubated with host represents 5 or 6 replicas.

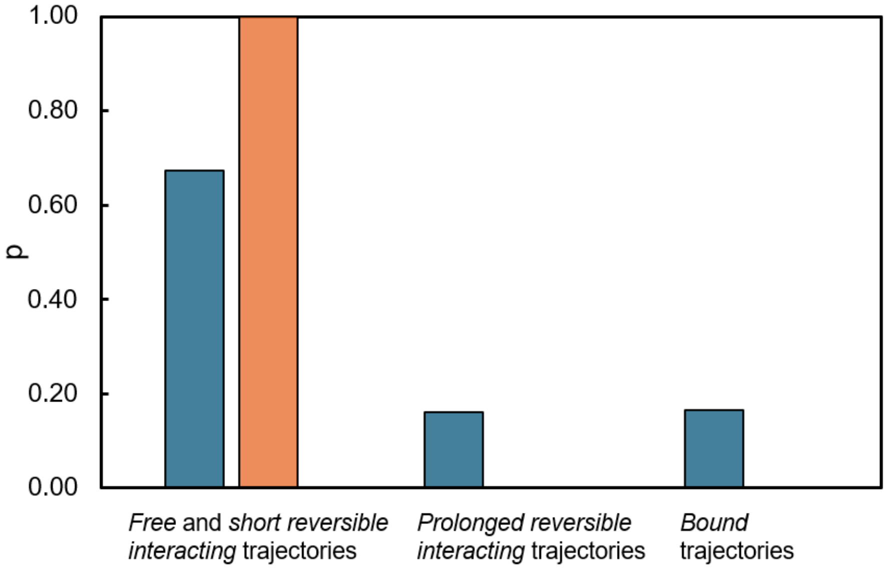

Supplementary figure 5 Proportion of *free* and *short reversible interacting* (< 2 seconds), *prolonged reversible interacting* (> 2 seconds), and *bound* trajectories in presence of *E. coli* B (blue; *n* = 673) and K12 mutant strain lacking the appropriate host receptors (orange; *n* ≈ 300).

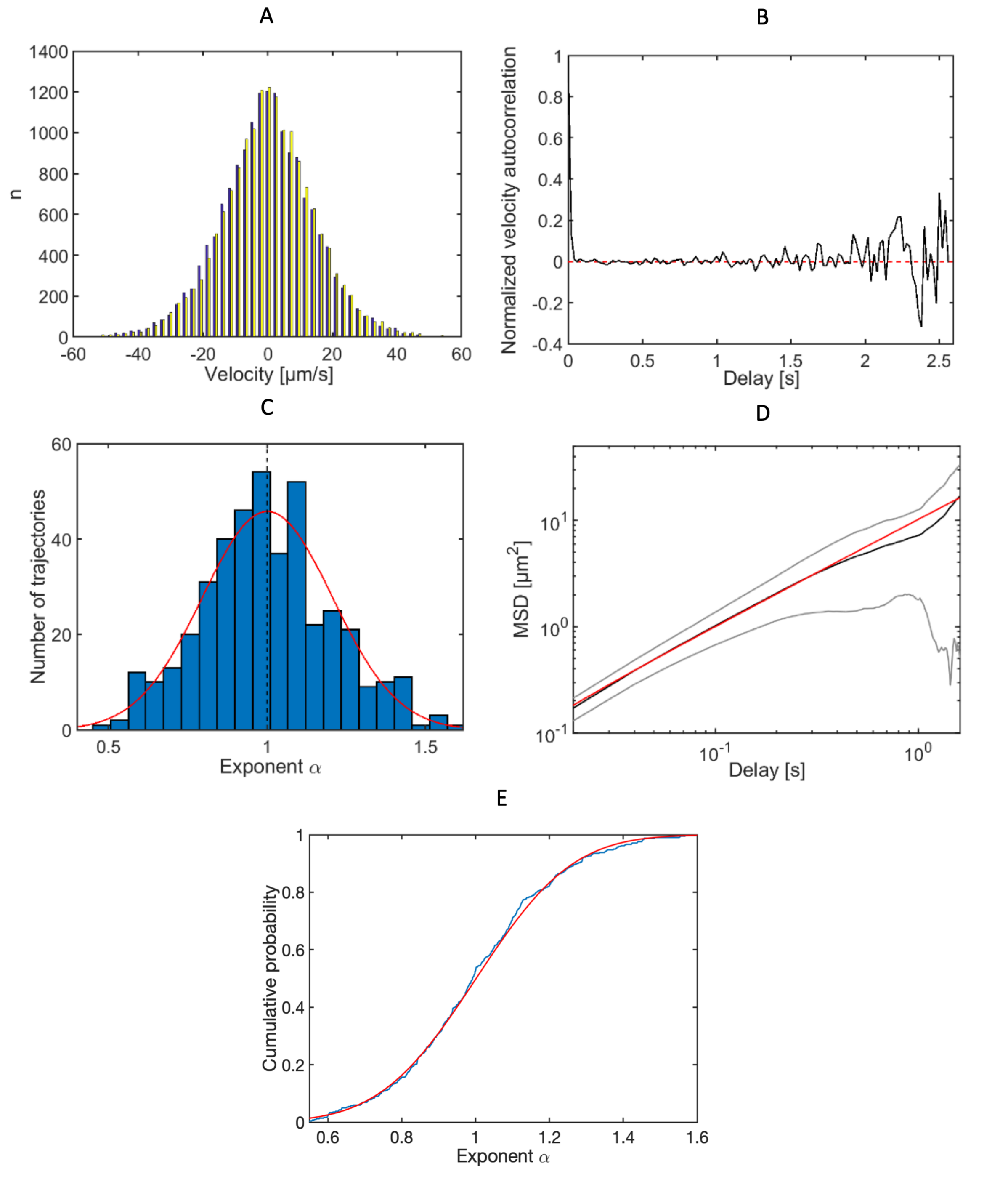

Supplementary figure 6 Freely diffusing phages exhibited random and uncorrelated displacement. A, Velocity analysis distribution for all individual trajectories classified within the group *free*. Yellow and purple bars presented *x* and *y* displacement, respectively. **B,** Normalized velocity autocorrelation. **C,** Histogram showing values for exponent α for each individual trajectory. The values for α were obtained by a linear fit on the first 25% of the log-log MSD curve as a function of delay time. The red curve presents a fit for normal distribution with sigma 1.00 and mu 0.21. The black dashed line presents the mean value of α. **D,** Diffusion coefficient estimation by means of a linear weighted fit (red line) on the first 15% of the weighted average of the MSD curve (black line). Area between the grey lines represents the weighted standard deviation over all individual MSD curves. **E,** The CDF was used to calculate the cumulative probability for the range of observed α exponent. Blue and red curves presented the empirical CDF and standard normal CDF with mu 1.00 and sigma 0.21, respectively.

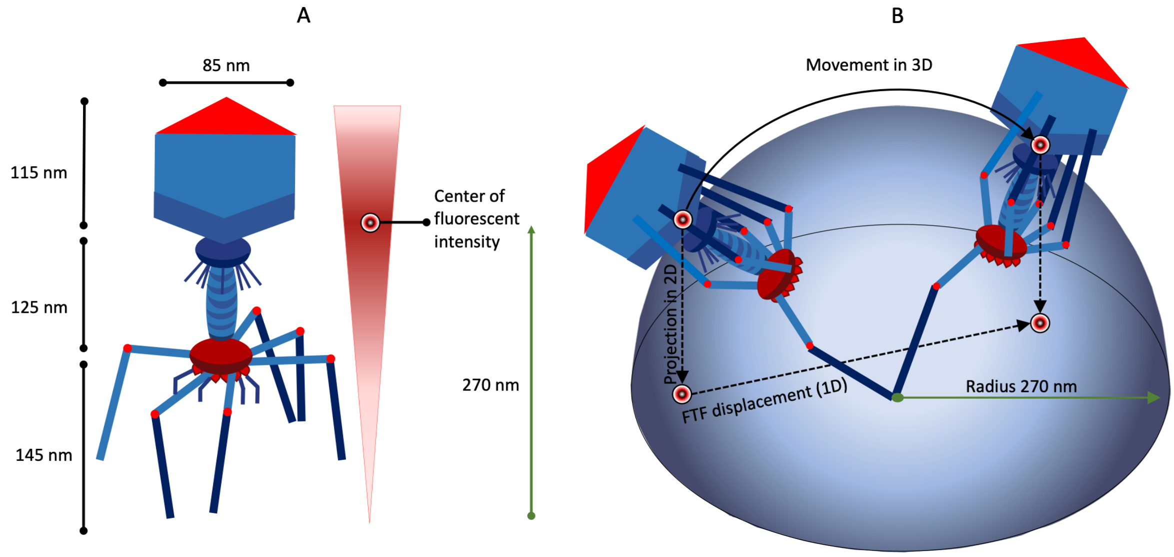

Supplementary figure 7 Modeled values for FTF displacement values of a T4 phage tethered to a surface with a single LTF. A, Dimensions of T4 phage components [1, 2] and fluorescence intensity across T4 phage based on density of labels on the phage. **B,** Range in which a fluorescently labeled T4 phage particle can be detected when tethered to a location on the surface with a single LTF.

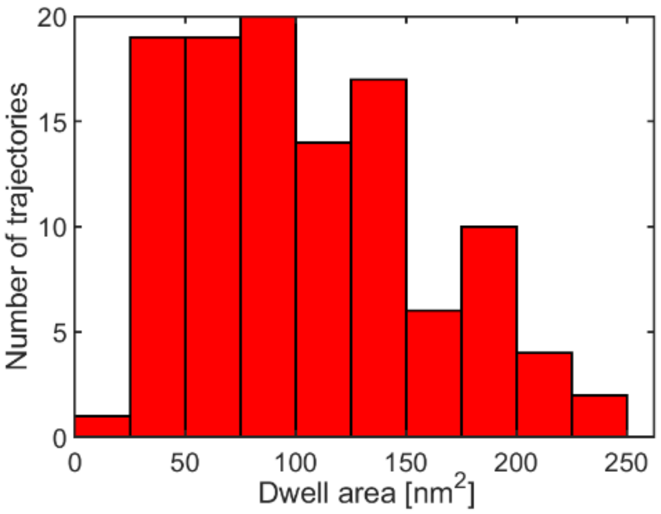

Supplementary figure 8 Dwell area of *bound* phages. Dwell area was obtained by calculating the surface area within the convex hull for each individual trajectory classified within the group of *bound* trajectories.

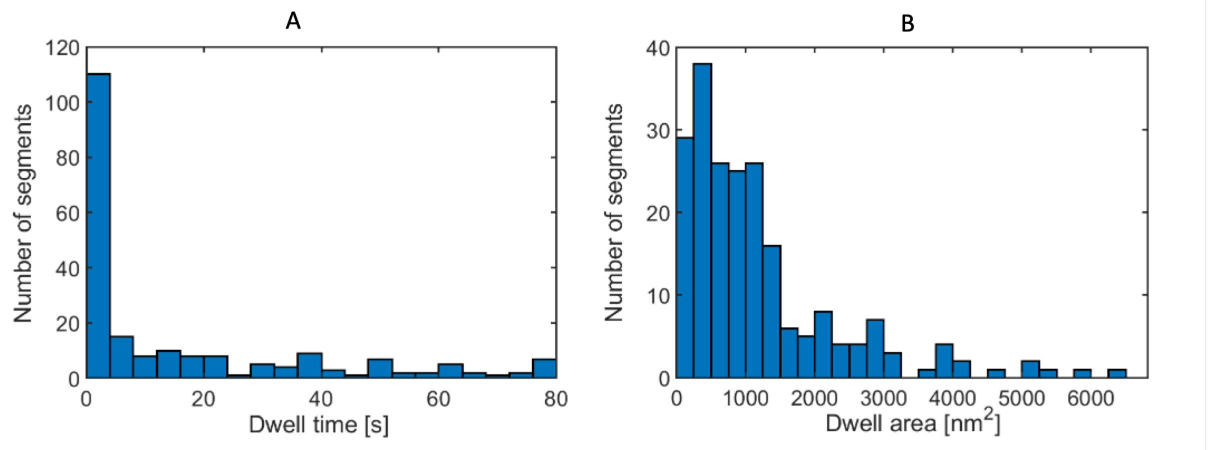

Supplementary figure 9 Characterization of tethered segments of *interacting* phage trajectories. A, Dwell time and **B,** dwell area of tethered segments that were obtained by calculating the duration and surface area within the convex hull for each tethered segment of individual trajectories identified as *interacting*.

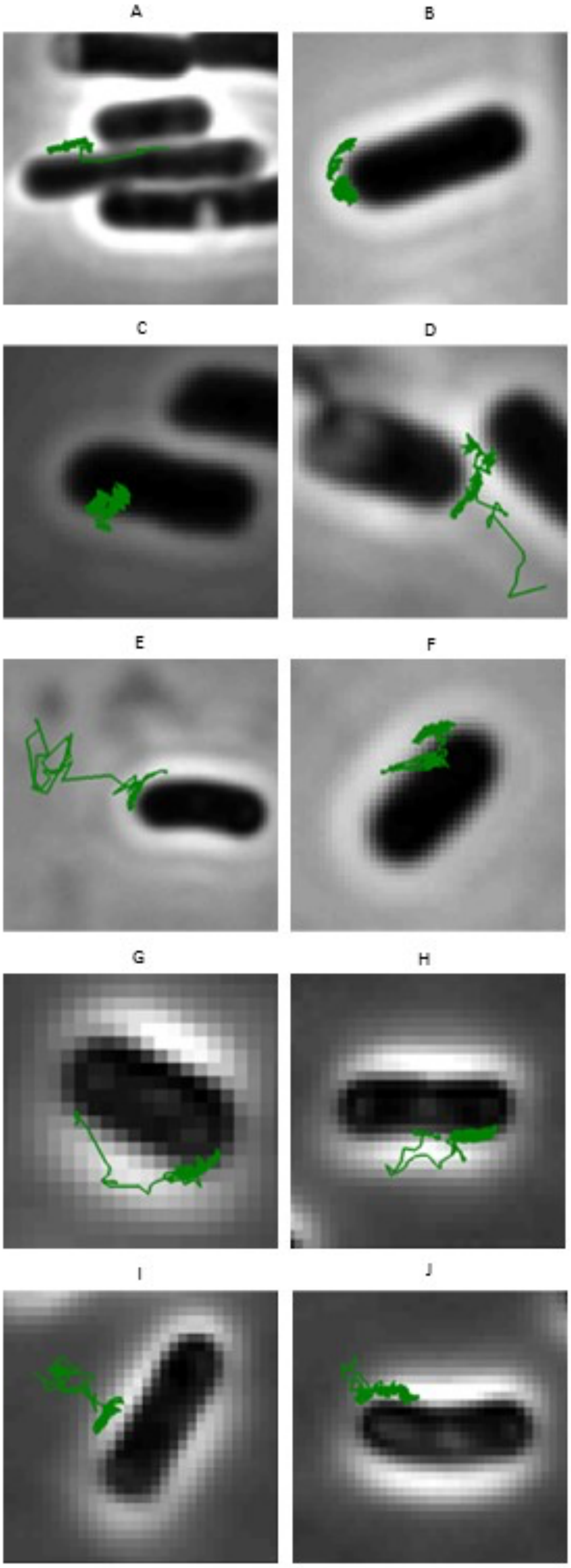

Supplementary figure 10 Interacting trajectories exceeding theoretical area accessible by a phage tethered to a single LTF at the cell surface. Images provide representative trajectories of observed behavior of T4 phage exploring a large area at the host cell surface of *E. coli* B.

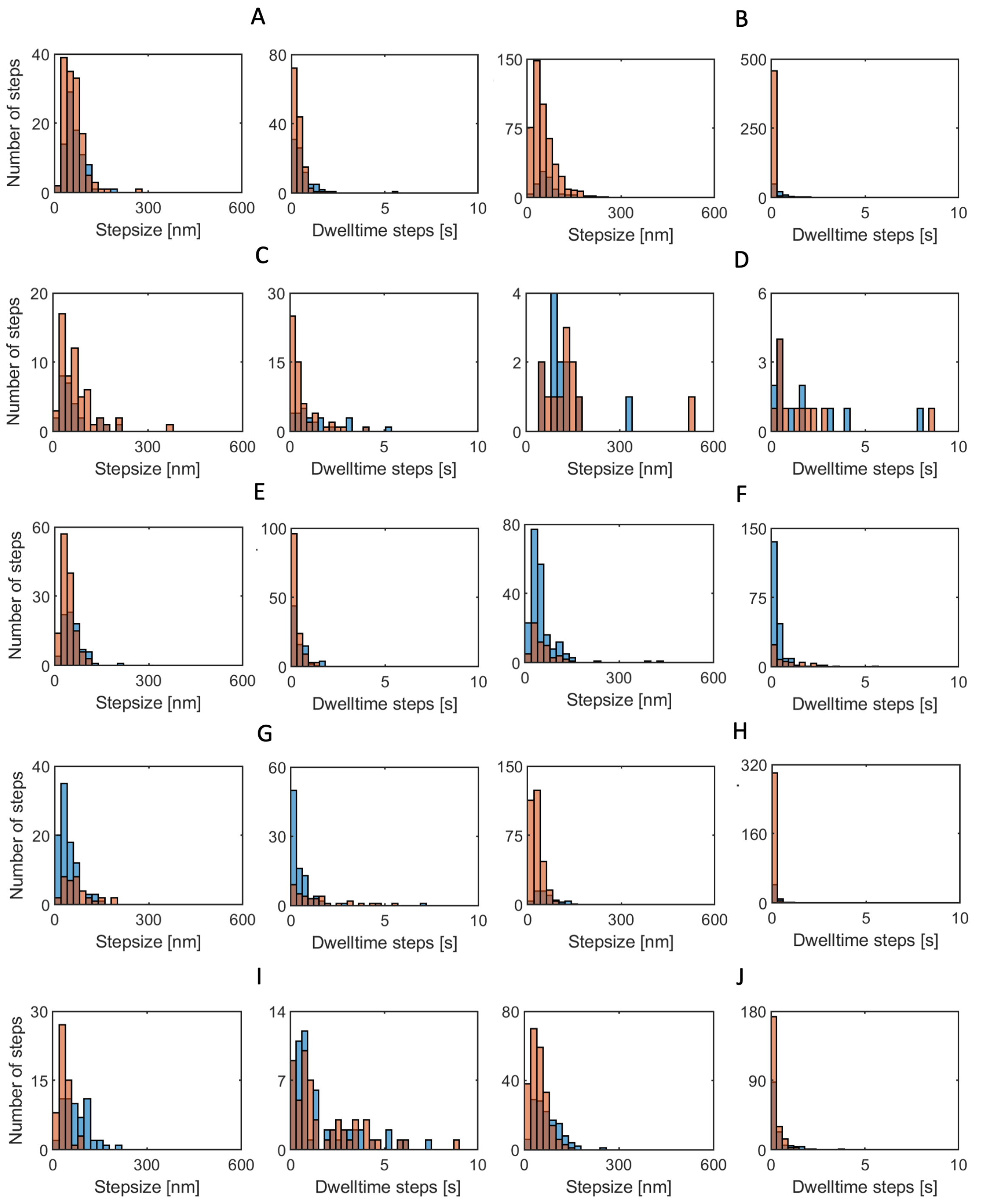

Supplementary figure 11 Interacting trajectories show steps. For each of the selected T4 trajectories, steps were fitted by the algorithm Stepfinder [6, 7] along one dimension, x- and y-axis. histogram of stepsizes. Right: histogram of dwelltime of steps. Data is obtained in 1D, were blue and red indicates stepsizes and dwelltime of steps in x- and y-direction, respectively. Data corresponds to tethered segments of interacting trajectories depicted in Supplementary figure 10.

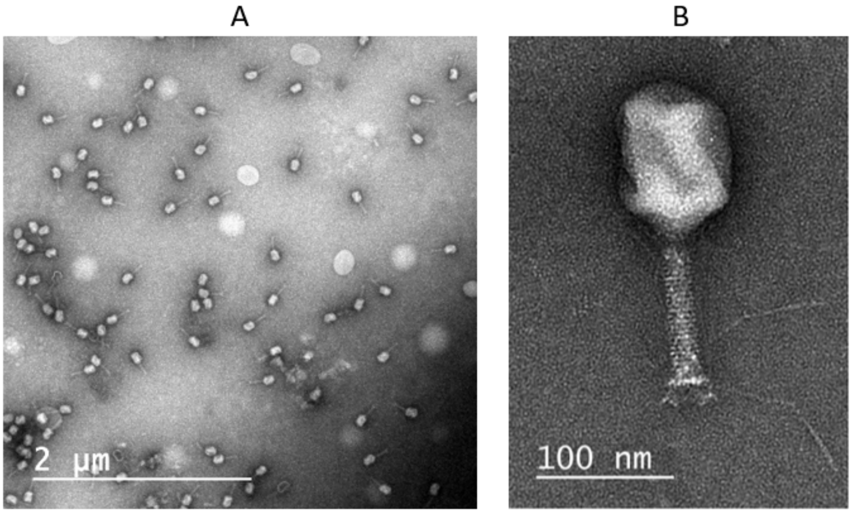

Supplementary figure 12 Electron micrographs of T4 phage. Negative staining TEM imaging of purified T4 phage before labeling shows that the majority of T4 phages are intact (non-contracted state) and that the phage suspension is free from large contamination of other proteins or debris (non-phage proteins).

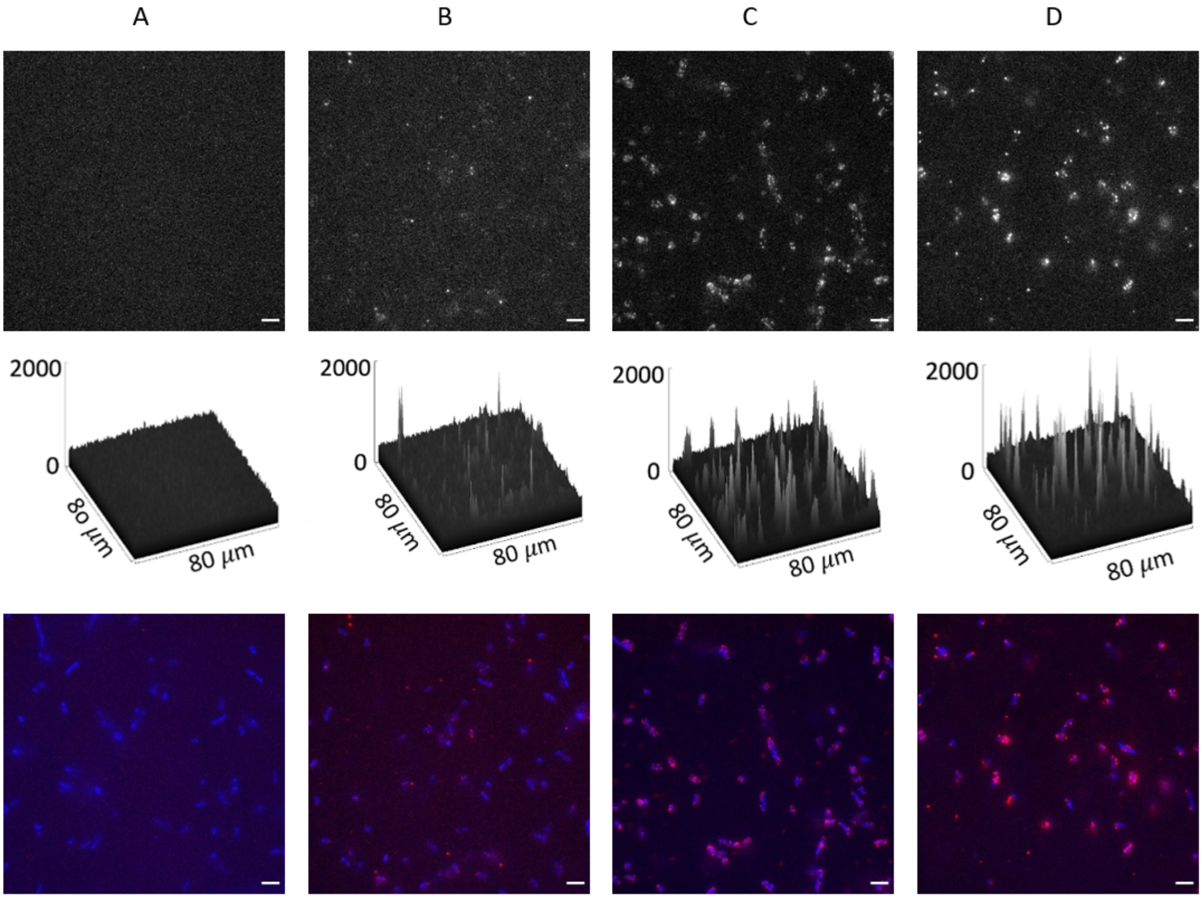

Supplementary figure 13 Effect of Alexa647 dye concentration on detectability of bacteriophage T4. T4 phages were incubated with four different concentrations of Alexa647 dye containing a reactive NHS-ester that binds to either exposed lysines or the N-termini, with **A,** 100 µM, **B,** 1 mM, **C,** 10 mM, **D,** 100 mM. Fluorescent images (top), their intensity profile (middle), and an overlay (bottom) of T4 (red) with *E. coli* B (blue, stained with DAPI). Scale bars represent 5 µM.

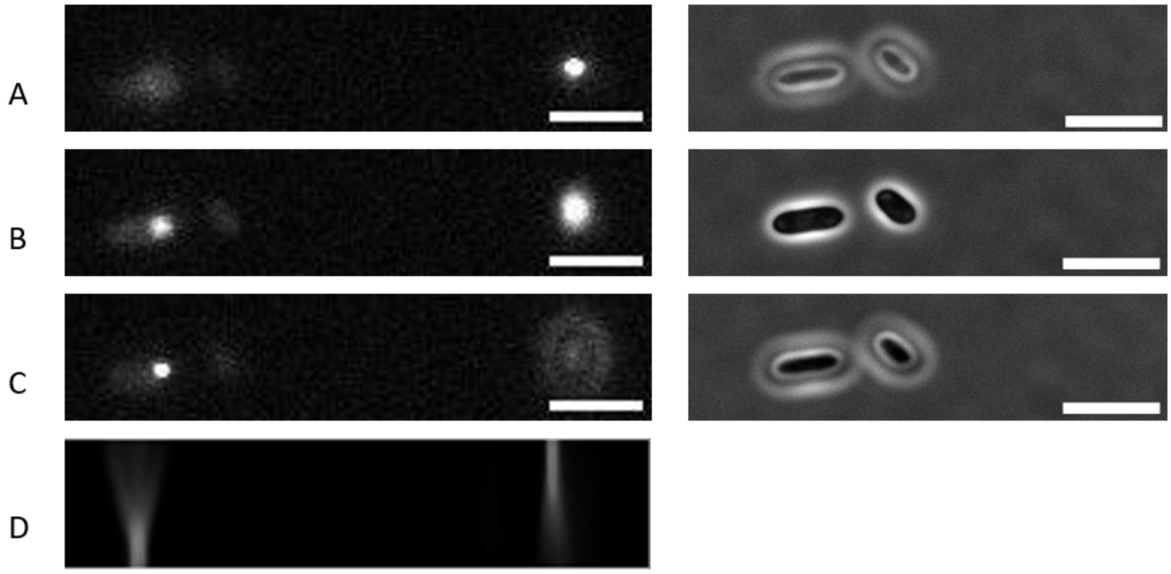

Supplementary figure 14 Phage detection outside the focal plane. Z-stack of the fluorescent channel (left) and corresponding phase contrast channel (right). Focus shifts from **A,** bottom of cell, **B,** mid-cell, **C,** top of cell. **D,** Intensity profile of fluorescent channel in *z*-direction showing a visual detection depth from the glass slide (attached T4 phage to glass surface) up to at least 1.5 µm above the focal plane height (bound T4 phage on top of cell). Scale bar represents 5 µM.

Supplementary table 1 Steps within interacting trajectories.

Trajectory	1D direction	Number of steps	Mean stepsize (nm) ± SD	Duration of on-cell segment (s)	Mean dwelltime of step (s) ± SD	Number of steps s^{-1}	Number of steps above conservative threshold of 50 nm
A	*x*	84	63.67 ± 29.83	49.88	0.59 ± 0.71	1.68	55
	y	137	60.57 ± 33.36	49.86	0.36 ± 033	2.75	78
B	*x*	89	64.74 ± 34.38	49.02	0.55 ± 1.19	1.82	58
	y	477	52.30 ± 38.28	49.92	0.10 ± 0.31	9.56	203
C	*x*	27	66.61 ± 49.76	37.80	1.40 ± 1.26	0.71	13
	y	58	74.68 ± 58.86	37.86	0.65 ± 0.81	1.53	33

D	*x*	12	121.27 ± 71.69	21.84	1.82 ± 2.20	0.55	12
	y	12	147.79 ± 118.82	20.64	1.72 ± 2.16	0.58	12
E	*x*	82	57.96 ± 31.72	34.48	0.42 ± 0.41	2.38	43
	y	135	42.62 ± 21.93	34.72	0.26 ± 0.28	3.89	43
F	*x*	203	51.08 ± 42.26	57.74	0.28 ± 0.29	3.52	71
	y	61	57.24 ± 53.48	57.08	0.94 ± 1.07	1.07	24
G	*x*	92	41.96 ± 28.82	49.82	0.54 ± 0.95	1.85	22
	y	36	71.53 ± 45.17	49.10	1.36 ± 1.44	0.46	21
H	*x*	56	57.47 ± 31.29	13.38	0.24 ± 0.39	1.12	27
	y	306	29.65 ± 18.95	13.45	0.04 ± 0.06	22.75	43
I	*x*	58	76.11 ± 40.57	96.07	1.66 ± 1.76	4.31	36
	y	54	36.96 ± 17.76	97.65	1.81 ± 1.81	4.01	9
J	*x*	132	70.98 ± 39.98	44.90	0.34 ± 0.43	9.81	87
	y	224	45.96 ± 26.72	45.11	0.20 ± 0.35	16.65	85

References

[1] M.L. Yap, M.G. Rossmann, Structure and function of bacteriophage T4, Future Microbiol 9(12) (2014) 1319-27.
[2] P.G. Leiman, P.R. Chipman, V.A. Kostyuchenko, V.V. Mesyanzhinov, M.G. Rossmann, Three-dimensional rearrangement of proteins in the tail of bacteriophage T4 on infection of its host, Cell 118(4) (2004) 419-29.
[3] N. Otsu, A Threshold Selection Method from Gray-Level Histograms, IEEE Transactions on Systems, Man, and Cybernetics 9(1) (1979) 62-66.
[4] S. van der Walt, J.L. Schonberger, J. Nunez-Iglesias, F. Boulogne, J.D. Warner, N. Yager, E. Gouillart, T. Yu, c. scikit-image, scikit-image: image processing in Python, PeerJ 2 (2014) e453.
[5] A.S. Ghosh, K.D. Young, Helical disposition of proteins and lipopolysaccharide in the outer membrane of Escherichia coli, J Bacteriol 187(6) (2005) 1913-22.
[6] J.W. Kerssemakers, E.L. Munteanu, L. Laan, T.L. Noetzel, M.E. Janson, M. Dogterom, Assembly dynamics of microtubules at molecular resolution, Nature 442(7103) (2006) 709-12.
[7] L. Loeff, J.W.J. Kerssemakers, C. Joo, C. Dekker, AutoStepfinder: A fast and automated step detection method for single-molecule analysis, Patterns (N Y) 2(5) (2021) 100256.
